# Bromophenols in Marine Algae and Their Bioactivities

**DOI:** 10.3390/md9071273

**Published:** 2011-07-22

**Authors:** Ming Liu, Poul Erik Hansen, Xiukun Lin

**Affiliations:** 1 Institute of Oceanology, Chinese Academy of Science, Qingdao 266071, China; E-Mail: lmouc@hotmail.com; 2 Department of Science, Systems and Models, Roskilde University, P.O. Box 260, DK-4000 Roskilde, Denmark

**Keywords:** bromophenols, marine algae, bioactivity

## Abstract

Marine algae contain various bromophenols that have been shown to possess a variety of biological activities, including antioxidant, antimicrobial, anticancer, anti-diabetic, and anti-thrombotic effects. Here, we briefly review the recent progress of these marine algal biomaterials, with respect to structure, bioactivities, and their potential application as pharmaceuticals.

## 1. Introduction

Marine algae are one of the richest sources of structurally diverse natural products. In recent years, an increasing number of novel compounds have been isolated from marine algae and many of them have been reported to possess interesting biological activities [1–3]. One kind of these marine algae derived compounds is the bromophenols (BPs). BPs share one or several benzene rings, a varying degree of bromine and hydroxyl-substituents (see schemes). The first two marine BPs were isolated from the red algae *Rhodomela larix* [4] and thereafter, many novel BPs were isolated and identified from diverse species of marine algae, including red algae [4–33], brown algae [34–39], and green algae [40–48]. It seems that species collected at low tide have a higher content of simple BPs [49]. Some, but not all, species of red algae have a relative higher content of certain BPs [49], which may possibly explain why so many BPs have been reported from red algae. The BPs were also found to exist in other lower marine organisms such as ascidians [50–52] and sponges [53–62]. BPs are common marine secondary metabolites, and biosynthesized in the presence of bromoperoxidases, hydrogen peroxide, and bromide [63,64]. The concentration of bromide is about 0.65 mg/kg in seawater and in marine algae [49]. The ecological function of BPs is not yet clear, but some of them may play a role in chemical defense and deterrence [65]. Recent studies revealed that the marine BPs exhibit a wide spectrum of beneficial biological activities [7,10,16,21,25,26,66–70], and therefore these novel BPs have attracted much attention in the field of functional food and pharmaceutical agents. In this mini-review, we focus on BPs from marine algae and present an overview of their bioactivities and potential application in pharmaceutics, since there are only a few reviews in this area [71].

## 2. Bioactivities of BPs and Potential Use in Medicine

### 2.1. Antioxidant Activity

Free radicals attack macromolecules (e.g., membrane lipids, proteins, enzymes, DNA, and RNA) and play a pivotal role in several health disorders such as cancer, diabetes, neurodegenerative and inflammatory diseases. Therefore, antioxidants may have a beneficial effect on human health by preventing free radical damage.

**Figure 1:**
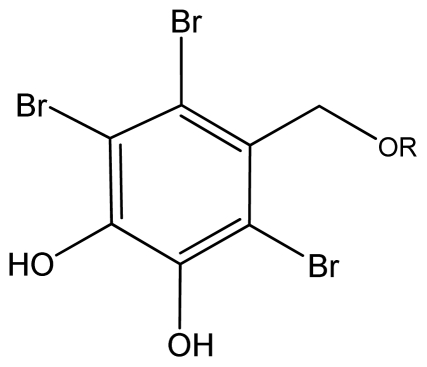
3,4-dihydroxy-2,5,6-tribromobenzyloxy unit.

**Scheme 1:**
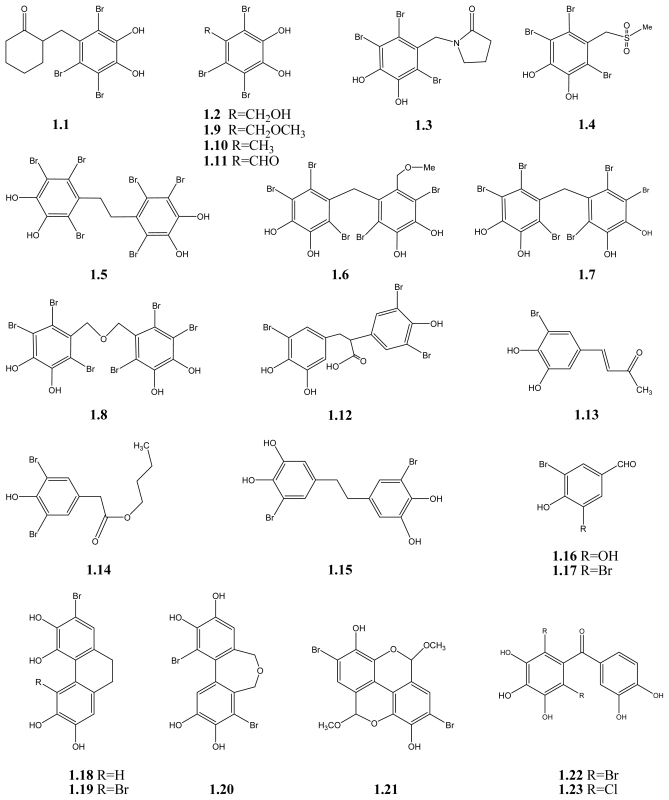
BPs with antioxidant activity.

A growing body of results indicates that BPs have potential antioxidant activity, mainly determined by the 1,1-diphenyl-2-picrylhydrazyl (DPPH) radical scavenging method. For example, BPs **1.1**–**1.11** (see Scheme 1) isolated from the red algae *Symphyocladia latiuscula*, were reported to possess DPPH radical scavenging activities [20,21]. All of these BPs are fully substituted by different groups and highly brominated, and many of them have a 3,4-dihydroxy-2,5,6-tribromobenzyloxy unit in the molecule. These BPs all show scavenging activity towards DPPH radical. Compound **1.2** has the highest activity, while compound **1.11** shows the lowest, with IC_50_ values of 7.5 and 24.7 μM, respectively [20,21]. Thus both are more potent than the positive control butylated hydroxytoluene (IC_50_ = 81.8 μM) (see also Table 1). It seems that their antioxidant activity may have a close connection to the number of hydroxyl groups in the molecule [21]. Moreover, their common 3,4-dihydroxy-2,5,6-tribromobenzyloxy unit or derivatives thereof (see Figure 1) may be another important factor for their activity.

A series of BPs isolated from the red algae *Polysiphonia urceolata* also exhibit significant antioxidant activity. These BPs (**1.12**–**1.21**, Scheme 1), are substituted to different degrees and all of them show scavenging activity towards DPPH radical. Compounds **1.18** and **1.19**, which both have four hydroxyl groups in the molecules, are the most active, with IC_50_ values of 6.8 and 6.1 μM, respectively. In contrast, compound **1.17**, which only has one hydroxyl substituent in the molecule, is the least active with an IC_50_ of 35.8 μM [25,26]. Therefore, this further supports the idea that the number of hydroxyl groups in the molecules plays a vital role for the antioxidant activity. Another important factor is conjugation (in the chemical sense) as seen by comparison of **1.19** with **1.15**. The former has conjugation in the dihydrophenanthrene skeleton. Conjugation effects can of course also be achieved by substituents like nitro, acetyl or aldehyde groups in *para*-position to the OH-group.

By comparing the IC_50_ of **1.5** and **1.15**, also **1.18** and **1.19**, it seems that bromination is not a determining factor. Bromination decreases the activity slightly in the case of **1.5** and **1.15**, whereas bromination increased it slightly for **1.19** *vs.* **1.18**. In another comparison between the natural BPs and their corresponding debrominated compounds, it was found that bromination also lead to a decrease in the antioxidant activity [73]. Therefore, bromination in the present BPs seems of little importance for their antioxidant activity. Recently, Li *et al.* have investigated some synthetic BPs (**1.22**), but they have only little activity [72]. However, **1.22** and **1.23** allow a comparison between bromine and chlorine substitution. Brominations lead to slightly more active compounds. A number of chlorinated compounds as well as reference compounds, compounds without halogens, have also been investigated [74]. It is obvious that the 1,4 dihydroxy arrangement is very suitable for antioxidant activity.

Up to now, about 30 BPs from marine algae were reported to have antioxidant activity *in vitro*. However, no *in vivo* antioxidant studies on marine BPs and their activity have been reported, and discussions on the structure and activity relationship (SAR) about BPs are sporadic [21,26,72,73]. Nevertheless, recent studies reveal BPs to be one of the potential candidates in the prevention of diseases related to free radical attack, such as cancer, diabetes, neurodegeneration, and inflammation.

### 2.2. Anticancer Activity

**Scheme 2:**
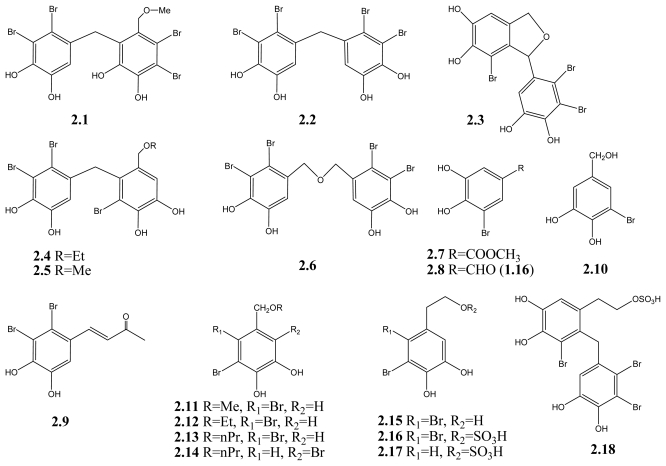
BPs with anticancer activity.

Chemotherapy is one of the major therapeutic approaches for cancer treatment, and several naturally obtained anticancer drugs, such as camptothecin and taxol, are used clinically [75]. It is believed that it is a promising strategy to screen naturally occurring compounds in order to discover novel anticancer agents. Several studies have reported that the marine BPs could inhibit the proliferation of a number of cancer cell lines *in vitro* and the growth of tumors *in vivo*. For example, BPs derivatives isolated from the brown algae *Leathesia nana*, **2.1**–**2.6** (Scheme 2), which also share the 2,3-dibromo-4,5-dihydroxybenzyl unit, are cytotoxic against a variety of human cancer cell lines (Table 2), including A549, BGC823, MCF-7, BEL-7402, HCT-8 [35,76]. The *Leathesia nana* extract, rich in BPs, could inhibit the growth of Sarcoma 180 tumors *in vivo* and improve the immune system remarkably [76], indicating its potential use in cancer treatment.

BPs with cytotoxic activities from the red algae and green algae are structurally simpler than those from the brown algae and most of them contain one benzene ring. 3-bromo-4,5-dihydroxy benzoic acid methyl ester (**2.7**) and 3-bromo-4,5-dihydroxy-benzaldehyde (**2.8**), isolated from marine red algae *Rhodomela confervoides*, are selectively cytotoxic against KB, Bel-7402, and A549 cells (IC_50_ ranging from 3.09 to 8.71 μg/mL (12.5–40.1 μM) [33], while lanosol butenone (**2.9**), isolated from the New Zealand marine red algae *Osmundaria colensoi*, is cytotoxic against human leukemia cells with an IC_50_ value of 8.0 μM [67]. Another compound similar to **2.7** and **2.8**, 3-bromo-4,5-dihydroxybenzylalcohol (**2.10**), which was isolated from the tropical green algae *Avrainvillea nigricans*, was reported to be cytotoxic to KB cells with IC_50_ = 8.9 μg/mL (47 μM) [45].

In another study, cytotoxicity of BPs (compounds **2.11**, **2.12**, and **2.13**) from the red algae *Polysiphonia lanosa* was evaluated and some derivatives were synthesized. Compounds **2.11**, **2.12**, and **2.13** show obvious cytotoxicity against DLD-1 and HCT-116 cell lines with IC_50_ ranging from 1.32 to 14.6 μM. The most active compound is the synthetic compound **2.14** (2,5-dibromo-3,4-dihydroxybenzyl *n*-propyl ether), which could significantly inhibit the proliferation of DLD-1 and HCT-116 cell lines (IC_50_ = 1.72 and 0.08 μM, respectively), and arrest the cell cycle of DLD-1 cells [69]. The preliminary SAR investigation considers that the activity is largely influenced by the number and position of bromine substituent, as well as the number of phenolic groups and side chains [69].

The phenylethanol and the phenylethanol sulfate BPs (**2.15**–**2.18**) show moderate cytotoxicity against several human cancer cell lines including HCT-8, Bel-7402, BGC-823, A549, and A2780 [16]. Comparing the IC_50_ values of **2.15** with that of **2.16**, which is the sulfated **2.15**, it seems that there is no obvious difference between their activities and the sulfate group is dispensable. However, the activity increases after bromination in **2.17**, suggesting the importance of Br in the anticancer activity. To confirm the role of Br and sulfate group in their anticancer activity, further experiments are needed.

Besides their promising cytotoxicty against various cancer cells, the selectivity of these BPs should be considered. This problem is also one of the main challenges for the current anticancer drugs. Some BPs are also cytotoxic to normal cell lines, such as the human embryo lung fibroblasts (HELF) [33,77], with low selectivity, which may make their application *in vivo* difficult. Therefore, structural modification is needed to enhance their activity and selectivity. The anticancer activity of these BPs is largely confined to the *in vitro* level and the mechanism of action is still unclear. As reviewed in Section 2.1, BPs are considered to be free radical scavengers to prevent oxidative damage, which is a vital factor in carcinogenesis. Whether this antioxidant activity is responsible for their anticancer activity has yet to be established. More SAR studies and *in vivo* tests are needed to modify and evaluate these lead compounds. However, the anticancer activity of BPs mentioned above shows that BPs should be considered as a possible group of anticancer candidates, and these promising lead compounds may interest scientists in the organic synthetic and oncology area.

### 2.3. Antimicrobial Activity

**Scheme 3:**
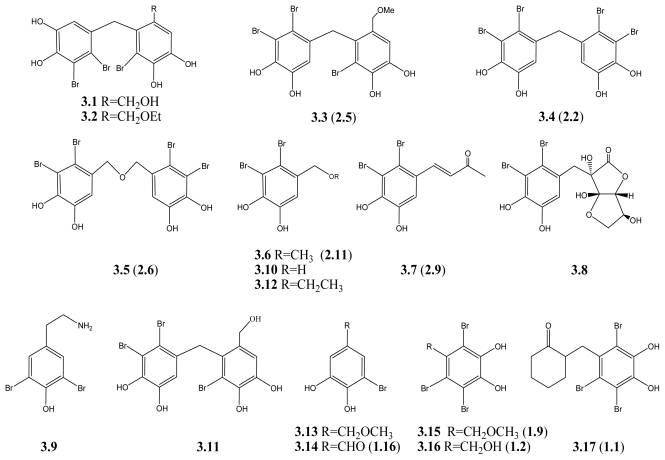
BPs with antimicrobial activity.

The discovery of novel antibacterial agents has been going on for many years. However, the new drugs have not kept pace with the increasing drug resistance of bacteria. One of the major challenges is the limitation of screening libraries [78,79]. Marine natural products may contribute to the improvement of these chemical libraries, and fortunately, various BPs from the marine algae have been reported to possess promising antibacterial activity. From the marine algae, *Rhodomela confervoides*, five BPs with antibacterial activity were isolated (**3.1**–**3.5**) (Scheme 3). Among these compounds, compound **3.5** has the most potent activity with the minimum inhibitory concentration (MIC) less than 70 μg/mL (121 μM), while compounds **3.2**, **3.3**, and **3.4** are moderately active, when tested against eight strains of Gram positive and Gram negative bacteria [66] (see Table 3). Another study showed that, lanosol methyl ether (**3.6**), lanosol butenone (**3.7**) and rhodomelol (**3.8**), isolated from the New Zealand red algae *Osmundaria colensoi*, all exhibit antibacterial activity against the MC155 strain of *Mycobacterium smegmatis* (IC_50_ 7.8, 26.2, and 28.1 μM, respectively) [67]. Lanosol ethyl ether (**3.12**) shows little antimicrobial activity, with mean bacteriostatic and fungistatic MIC of 0.27 ± 0.07 mg/mL (about 828 μM), and mean bacteriocidal and fungicidal MIC of 0.69 ± 0.15 mg/mL (about 2118 μM). Lanosol ethyl ether is thus a compound with good bacteriostatic and fungistatic activity. It has lower bactericidal and fungicidal activity but causes deformities in bacterial cells [32]. All these results indicate that these BPs would be potential lead compounds for antibacterial drug design. However, the SAR results are difficult to compare since the bacteria strains are not the same in different experiments.

In addition to the activity against bacteria, **3.3**, **3.4**, and **3.6** together with **3.9**, **3.10**, and **3.11** (see Scheme 2) may be promising candidates for antifungal agents in crop protection. These BPs could reduce the appressorium formation by the fungus *Magnaporthe grisea* on rice plants, due to inhibition of the isocitrate lyase (ICL). ICL plays an important role in the glyoxylate cycle and is highly expressed during appressorium-mediated plant infection [68]. The preliminary SAR reveals that the diphenylmethane skeleton and bromine moiety of BPs are essential for the ICL inhibition [68]. In subsequent experiments, series of BPs derivatives with different linkages between two phenol units and different bromination, were synthesized. The results reveal that these synthetic BPs derivatives also show strong inhibition against ICL and antimicrobial activity. The ICL enzyme inhibition activity increases with the increasing number of bromines in each series of molecules [80–82], suggesting the indispensible role of bromine in this enzyme inhibition.

The latest research found that 3-bromo-4,5-dihydroxybenzyl methyl ether (**3.13**) and 3-bromo-4,5-dihydroxybenzaldehyde (**3.14**), may be potential agents against fish pathogenic virus, infectious hematopoietic necrosis virus, and infectious pancreatic necrosis virus [83]. For the human herpetic infection, compounds **3.15**–**3.17** are considered as novel antiviral agents against wildtype herpes simplex type 1 (HSV-1), phosphonoacetic acid-resistant HSV-1 (AP^r^ HSV-1), and thymidine kinase deficient HSV-1 (TK^−^HSV-1) strains. IC_50_ values of **3.15** against these virus strains were reported as 3.02, 0.91, and 1.41 μg/mL, respectively. Oral administration (20 mg/kg) of **3.15** for 6–10 days could significantly delay the appearance of skin lesions and suppress the number of virus particles in the brain and skin without being toxic in HSV-1 strain 7401H infected mice [84]. Therefore, these reports show that certain BPs could be developed as promising antiviral agents.

An increasing number of BPs have been reported to exhibit antimicrobial activity. Many of them are highly brominated and share the same 3-bromo-4,5-dihydroxybenzyl unit (**3.1**–**3.8**, **3.10**–**3.12**, **3.13**–**3.17**), indicating the importance of the bromination and the 3-bromo-4,5-dihydroxybenzyl unit in the molecules. However, further SAR studies and structural modification are necessary to develop a new antimicrobial agent. Besides, the mechanisms underlying their antimicrobial activity, their activities as well as their toxicity *in vivo*, also have yet to be investigated. The increasing number of antimicrobial BPs is promising to the development of new antimicrobial agents.

### 2.4. Anti-Diabetic Activity

Despite the wide range of current anti-diabetic drugs used clinically, a large number of type 2 diabetes mellitus (T2DM) patients still suffer hyperglycemia and serious complications. Therefore, the therapeutic challenge of T2DM makes it necessary to discover new anti-diabetic agents.

**Scheme 4:**
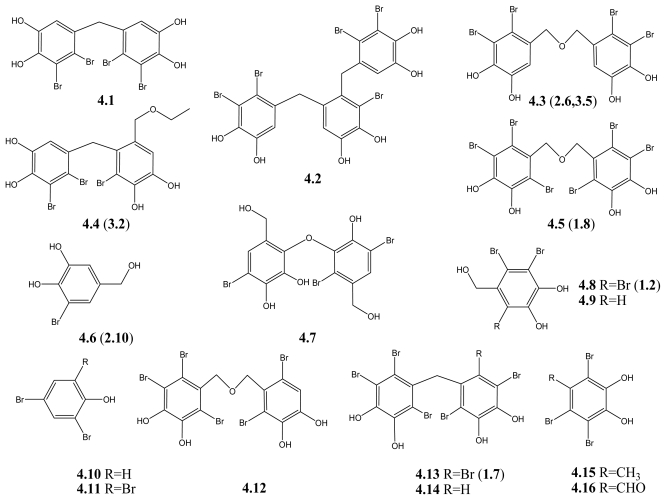
BPs with anti-diabetic activity.

Marine algae have been used for a long time as a remedy for diabetes in folk medicine [85]. Recently, BPs isolated from marine algae have been reported to be potential anti-diabetic agents, acting as both PTP1B inhibitors and alpha-glucosidase inhibitors. PTP1B regulates the insulin signaling pathway and agents targeting it could be effective in the treatment of diabetes [86]. Alpha-glucosidase is an enzyme that plays a central role in carbohydrate digestion and is a preferred target for anti-diabetic drugs. Bromophenol derivatives from red algae *Rhodomela confervoides*, **4.1**–**4.4** (Scheme 4), which contain one or two 2,3-dibromo-4,5-dihydroxybenzyl unit and highly brominated, inhibit PTP1B activity (IC_50_ were 2.4, 1.7, 1.5, and 0.84 μM, respectively) and the *R. confervoides* extracts could decrease the blood glucose level in diabetic rats [70]. These studies indicate that the *in vivo* anti-hyperglycemic activity could be partially due to the PTP1B inhibition. Recently, three analogs of **4.4** have been synthesized and investigated [87].

Moreover, other studies suggest that **4.3** together with **4.5** to **4.11** (Scheme 4) may be a novel kind of α-glucosidase inhibitor [7,8,10,11]. The most potent α-glucosidase inhibitor in the present series of BPs is bis (2,3,6-tribromo-4,5-dihydroxybenzyl) ether (**4.5**), with an IC_50_ of 0.03 μM [8], while the weakest one is 2,4-dibromophenol (**4.10**), with an IC_50_ of 110.4 μM. We suppose that the degree of bromination in these molecules may have a close relationship with their α-glucosidase inhibition based on the following: their IC_50_ values decrease with the increase of the bromine number in the molecules, for example, 3-bromo-4,5-dihydroxybenzyl alcohol (**4.6**) inhibits against α-glucosidase with IC_50_ of 100 μM, when one more position is brominated (**4.9**), the IC_50_ value decreases to 89 μM, while fully brominated, like in **4.8**, it is 11 μM (Table 4). The same phenomenon was observed in **4.3** and its corresponding compound **4.5**, in **4.10** and its corresponding compound **4.11**. In addition, it seems that the number of the phenyl units in the molecules also affect the enzyme inhibition activity, **4.3** and **4.5**, which possess diphenyl unit are much more active than the compounds with one phenyl unit (**4.6**, **4.8**, **4.9**). The reason for these changes in activity remains unclear.

Besides inhibition against PTP1B and α-glucosidase, some BPs also inhibit the aldose reductase. Aldose reductase is the first enzyme of the polyol pathway, which is responsible for fructose formation from glucose and plays an important role in the development of degenerative complications of diabetes [88]. For example, BPs from the red algae *Symphyocladia latiuscula* **4.12**–**4.16** (Scheme 4), are reported to have aldose reductase inhibitory activity and could be used in the treatment of complications of diabetes, such as eye and nerve damage in T2DM patients [31].

BPs with strong alpha-glucosidase inhibition activity are being synthesized in our lab to evaluate their hypoglycemic activity *in vivo*, and we find that besides its antibacterial and antitumor activities, the alpha-glucosidase inhibitor, bis (2,3-dibromo-4,5-dihydroxybenzyl) ether (**4.3**) could also significantly alleviate the postprandial blood glucose level both in normal and diabetic animals [89]. Considering that alpha-glucosidase and PTP1B also play an important role in cancer, it seems useful to investigate if the alpha-glucosidase and PTP1B inhibition of BPs is relevant for their anticancer activity. PTP1B and alpha-glucosidase inhibition, together with the antioxidant activity mentioned in Section 2.1, suggest that BPs may be promising candidates for development of anti-diabetic agents.

### 2.5. Other Bioactivities

Besides the activities mentioned above, other bioactivities were also reported, including the inhibition against thrombin, 3-hydroxy-3-methylglutaryl coenzyme A (HMG-CoA) reductase, and phospholipase A_2_.

**Scheme 5:**
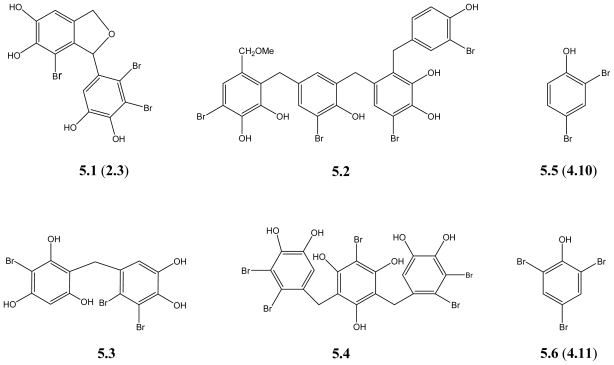
BPs with other activities.

Thrombin is the ultimate proteinase of the coagulation cascade, which makes it an attractive target for the treatment of a variety of cardiovascular diseases. A bromophenol derivative (+)-3-(2,3-dibromo-4,5-dihydroxyphenyl)-4-bromo-5,6-dihydroxy-1,3-dihydro-isobenzofuran (**5.1**) (Scheme 5), isolated from the brown algae *Leathesia nana* exhibits significant thrombin inhibitory activity both *in vitro* and *in vivo* [38,90].

Rawsonol (**5.2**), a novel brominated diphenyl methane derivative, which is isolated from the green algae *Avrainvillea rawsoni*, inhibits the activity of HMG-CoA reductase [41]. HMG-CoA reductase is the key enzyme in cholesterol biosynthesis and the target of the widely available cholesterol-lowering drugs. The observed inhibition therefore indicates the potential use of diphenylmethane derivatives in lowering of cholesterol levels.

Moreover, anti-inflammatory effects were observed for Vidalols A (**5.3**) and B (**5.4**), which are obtained from the red alga *Vidalia obtusaloba*. The two compounds could clearly reduce the edema in phorbol ester induced swelling of the mouse ear, via inhibition of the arachidonic acid metabolic pathway enzyme phospholipase A_2_ (bee venom PLA_2_) [29]. Therefore, Vidalols A and B could be possible lead compounds for the design of anti-inflammatory agents.

However, not all the BPs show beneficial health effects. Some natural BPs such as 2,4-dibromophenol (**5.5**) and 2,4,6-tribromophenol (**5.6**), used as flame retardants and fungicides, are suspected to show negative impact on human and animal health [91,92]. For instance, **5.5** is revealed to bind to the estrogen receptor and act as an endocrine disruptor [91], while **5.6** inhibits cell proliferation and induces neuronal cell differentiation in human neuroblastoma cell line (SH-SY5Y) [93]. Another study reveals that **5.5** and **5.6** disturb cellular Ca^2+^ signaling in neuroendocrine cells (PC12) [94]. **5.6** tested *in vivo* is reported to interfere with the steroidogenic pathway, reproduction, and embryo development in zebrafish [95,96]. Besides **5.6**, other brominated indoles and phenols are also reported to be toxic (lethal and malformations) to the zebrafish embryos [97]. These results indicate the possible toxicity of **5.5** and **5.6** both *in vitro* and *in vivo*. Therefore, their possible toxicity for all life forms should be kept in mind.

## 3. Conclusions

The BPs obtained from macroalgae enlarge the chemical library and improve the opportunity to discover new pharmaceutical agents, and interesting novel BPs will still be found in the future. One interesting point is that no BPs were reported in microalgae. The exact mechanism is still unknown underlying the distribution of BPs. Further study is needed to address if BPs could be isolated from microalgae. Intensive efforts and obvious progress have been made in recent years and provide evidence that BPs exhibit diverse biological activities, including antioxidant, antibacterial, anticancer, and anti-diabetic activity. SAR study reveals that some core structure or substituents may play a critical role for the biological activity of BPs. In some cases like antioxidant effect, the presence of bromine substituents seems of little importance, whereas the number of hydroxyl groups is clearly important. The mutual orientations of the hydroxyl groups are also a useful character for antioxidant activity. Although *para*-substitution of hydroxyl groups is considered the most effective mutual orientations, this kind of mutual orientation was not found in BPs. *Ortho*-substitution is also a useful character for antioxidant activity. In both cases quinone formation is easy. In addition, conjugation connected to substituents such as acetyl, nitro or phenyl is important for antioxidant activity. Bromines play a key role in the anti-diabetic activity and cytotoxicity. With regard to toxicity towards cancer cells, the number of bromines plays a role and alkylation of phenol groups lower the activity. It should also be noted that the activity towards diverse cell lines differs. However, too few derivatives are available to draw a conclusion about the importance of the relative positions of substituents. A structure such as that found in Figure 1, or a derivative thereof, seems to be common for the biological effects. The mode of action of BPs has not well been documented, but it should be kept in mind that bromine may act as a halogen bond donor as seen in 4,5,6,7-tetrabromobenzotriazole [98].

Unfortunately, so far it has proved difficult to identify a selective, safe and effective new drug from these BPs. One of the major challenges in developing potential therapeutic agents from BPs is the limited amount of BPs in these marine algae, which hinder an immediate *in vivo* investigation. Another challenge seems to be that the present research mainly focuses on the isolation and characterization of BPs compounds, but pays less attention to the biological activities, the mechanisms underlying their activities, and the structure–activity relationships. Therefore, more pharmaceutical chemistry including synthesis of compounds and *in vivo* studies are needed in order to develop novel agents.

## Figures and Tables

**Table 1:** IC_50_ of the DPPH scavenging activity and names of compounds in Scheme 1.

No.	IC_50_ (μM)	Names
**1.1**	8.5	(2*R*)-2-(2,3,6-tribromo-4,5-dihydroxybenzyl)-cyclohexanone [20]
**1.2**	7.5	2,3,6-tribromo-4,5-dihydroxybenzylalcohol [20]
**1.3**	18.5	1-(2,3,6-tribromo-4,5-dihydroxybenzyl)pyrrolidin-2-one [21]
**1.4**	24	2,3,6-tribromo-4,5-dihydroxybenzyl methyl sulfone [21]
**1.5**	10.2	1,2-bis(2,3,6-tribromo-4,5-dihydroxyphenyl)ethane [21]
**1.6**	10.5	6-(2,3,6-tribromo-4,5-dihydroxybenzyl)-2,5-dibromo-3,4-dihydroxybenzyl methyl ether [21]
**1.7**	8.1	Bis(2,3,6-tribromo-4,5-dihydroxyphenyl)methane [21]
**1.8**	8.5	Bis(2,3,6-tribromo-4,5-dihydroxybenzyl)ether [21]
**1.9**	15.5	2,3,6-tribromo-4,5-dihydroxybenzyl methyl ether [21]
**1.10**	14.0	2,3,6-tribromo-4,5-dihydroxymethylbenzene [21]
**1.11**	24.7	2,3,6-tribromo-4,5-dihydroxybenzaldehyde [21]
**1.12**	21.9 ± 0.1	3-(3-bromo-4,5-dihydroxyphenyl)-2-(3,5-dibromo-4-hydroxyphenyl) propionic acid [25]
**1.13**	9.67 ± 0.04	(*E*)-4-(3-bromo-4,5-dihydroxyphenyl)-but-3-en-2-one [25]
**1.14**	16.11 ± 0.06	(3,5-dibromo-4-hydroxyphenyl) acetic acid butyl ester [25]
**1.15**	19.64 ± 0.09	1,2-bis(3-bromo-4,5-dihydroxyphenyl)ethane [25]
**1.16**	20.3	3-bromo-4,5-dihydroxybenzaldehyde [26]
**1.17**	35.8	3,5-dibromo-4-hydroxybenzaldehyde [26]
**1.18**	6.8	7-bromo-9,10-dihydrophenanthrene-2,3,5,6-tetraol [26]
**1.19**	6.1	4,7-dibromo-9,10-dihydrophenanthrene-2,3,5,6-tetraol [26]
**1.20**	8.1	1,8-dibromo-5,7-dihydrodibenzo[c,e]oxepine-2,3,9,10-tetraol [26]
**1.21**	15.1	Urceolatol [26]
**1.22**	96.2	2,6-dibromo-3,3′,4,4′,5-pentahydroxydiphenylmethanone [72]
**1.23**	87.3	2,6-dichloro-3,3′,4,4′,5-pentahydroxydiphenylmethanone [72]

**Table 2:** Anticancer activity and names of compounds in Scheme 2.

No.	IC_50_ and cells	Names
**2.1**	2.5 (A549), 8.8 (BGC823) 2.7 (MCF-7), 4.8 (Bel7402) 16.8 (HCT-8)	6-(2,3-dibromo-4,5-dihydroxybenzyl)-2,3-dibromo-4,5-dihydroxy benzyl methyl ether [35]
**2.2**	1.8 (A549), 3.8 (BGC823) 2.7 (MCF-7), 2.2 (HCT-8) >18.2 (Bel7402)	2,2′,3,3′-tetrabromo-4,4′,5,5′-tetrahydroxydiphenylmethane [35]
**2.3**	8.27 (MCF-7) 6.36 (HT-1080), μg/mL	(+)-3-(2,3-dibromo-4,5-dihydroxyphenyl)-4-bromo-5,6- dihydroxy-1,3-dihydroisobenzofuran [76]
**2.4**	>19 (A549), 4.6 (BGC823) 3.4 (MCF-7), 5.5 (Bel7402) 2.8 (HCT-8)	2,2′,3-tribromo-3′,4,4′,5-tetrahydroxy-6′-ethyloxymethyldiphenylmethane [35]
**2.5**	>19.5 (A549), 8.6 (BGC823) 21.4 (MCF-7), 20.7 (HCT-8) >1.9 (Bel7402)	3-bromo-4-(2,3-dibromo-4,5-dihydroxybenzyl)-5- methoxymethylpyrocatechol [35]
**2.6**	5.4 (A549), 18 (BGC823) 4.6 (MCF-7), 7.4 (Bel7402) 5.9 (HCT-8)	Bis(2,3-dibromo-4,5-dihydroxybenzyl)ether [35]
**2.7**	3.09 (KB), 3.18 (Bel-7402) 3.54 (A549), μg/mL	3-bromo-4,5-dihydroxybenzoic acid methyl ester [33]
**2.8**	8.71 (KB), 5.36 (Bel-7402) 7.56 (A549), μg/mL	3-bromo-4,5-dihydroxybenzaldehyde [33]
**2.9**	8.0 (HL-60)	Lanosol butenone [67]
**2.10**	47 (KB)	3-bromo-4,5-dihydroxybenzylalcohol [45]
**2.11**	14.6 ± 3.1 (DLD-1) 14.1 ± 2.5 (HCT116)	Lanosol methyl ether [69]
**2.12**	13.5 ± 2.3 (DLD-1) 2.51 ± 0.95 (HCT116)	Lanosol ethyl ether [69]
**2.13**	12.4 ± 1.1 (DLD-1) 1.32 ± 0.3 (HCT116)	Lanosol *n*-propyl ether [69]
**2.14**	1.72 ± 0.29 (DLD-1) 0.8 ± 0.63 (HCT116)	2,5-dibromo-3,4-dihydroxybenzyl *n*-propyl ether [69]
**2.15**	19.7 (A549), 19.9 (A2780) 19.4 (Bel-7402), 15.4 (HCT-8) 20.2 (BGC-823)	2,3-dibromo-4,5-dihydroxyphenylethanol [16]
**2.16**	14.7 (A549), 9.4 (A2780) 14.8 (Bel-7402), 14.0 (BGC-823) 14.6 (HCT-8)	2,3-dibromo-4,5-dihydroxyphenylethanol sulfate [16]
**2.17**	18.5 (A549), 20.8 (A2780) 20.4 (Bel-7402), 19.1 (BGC-823) 18.8 (HCT-8)	3-bromo-4,5-dihydroxyphenylethanol sulfate [16]
**2.18**	14.5 (A549), >16.9 (A2780) 13.5 (Bel-7402), 15.1 (BGC-823) 12.1 (HCT-8)	3-bromo-2-(2,3-dibromo-4,5-dihydroxybenzyl)-4,5- dihydroxyphenyethanol sulfate [16]

Notes: unit for IC_50_ is μM, unless labeled as μg/mL.

**Table 3:** Antimicrobial activity and names of compounds in Scheme 3.

No.	MIC/IC_50_ and Microbe	Names
**3.1**	MIC 140 μg/mL (a,b,c)	3-bromo-4-(2,3-dibromo-4,5-dihydroxyphenyl) methyl-5-(hydroxymethyl)-1,2-benzenediol [66]
**3.2**	MIC 70 μg/mL (a) MIC 140 μg/mL (b,c,d,e)	3-bromo-4-(2,3-dibromo-4,5-dihydroxyphenyl) methyl-5-(ethoxymethyl)-1,2-benzenediol [66]
**3.3**	MIC 70 μg/mL (a,b,c,d) IC_50_ 2.1 ± 0.1 μM (ICL)	3-bromo-4-(2,3-dibromo-4,5-dihydroxyphenyl) methyl-5-(methoxymethyl)-1,2-benzenediol [66,68]
**3.4**	MIC 70 μg/mL (a–g) IC_50_ 2.0 ± 0.1 μM (ICL)	4,4′-methylenebis(5,6-dibromo-1,2-benzenediol) [66,68]
**3.5**	MIC 70 μg/mL (a,b,f,g) MIC 140 μg/mL (d,e) MIC 35 μg/mL (c)	Bis(2,3-dibromo-4,5-dihydroxybenzyl)ether [66]
**3.6**	IC_50_ 125.6 ± 8.6 μM (ICL) IC_50_ 7.8 μM (h)	Lanosol methyl ether [67,68]
**3.7**	IC_50_ 26.2 μM (h)	Lanosol butanone [67]
**3.8**	IC_50_ 28.1 μM (h)	Rhodomelol [67]
**3.9**	IC_50_ 116.1 ± 7.3 μM (ICL)	3,5-dibromo-4-hydroxyphenylethylamine [68]
**3.10**	IC_50_ 92.6 ± 5.8 μM (ICL)	2,3-dibromo-4,5-dihydroxybenzylalcohol [68]
**3.11**	IC_50_ 2.8 ± 0.2 μM (ICL)	2,2′,3-tribromo-3′,4,4′,5-tetrahydroxy-6′-hydroxymethyl diphenylmethane [68]
**3.12**	MIC 0.69 ± 0.15 μg/mL (i) MIC 0.27 ± 0.07 μg/mL (j)	Lanosol ethyl ether [32]
**3.13**	IC_50_ 27 ± 6.3 μM (IHNV) 22.0 ± 0.6 μM (IPNV)	3-bromo-4,5-dihydroxybenzyl methyl ether [83]
**3.14**	IC_50_ 45 ± 9.1 μM (IHNV) 57.0 ± 10.6 μM (IPNV)	3-bromo-4,5-dihydroxybenzaldehyde [83]
**3.15**	IC_50_ 3.02 μg/mL (HSV-1) 0.91 μg/mL (AP^r^ HSV-1) 1.41 μg/mL (TK^−^HSV-1)	2,3,6-tribromo-4,5-dihydroxybenzyl methyl ether [84]
**3.16**	IC_50_ 7.82 μg/mL (HSV-1) 7.20 μg/mL (AP^r^ HSV-1) 11.21 μg/mL (TK^−^HSV-1)	2,3,6-tribromo-4,5-dihydroxybenzylalcohol [84]
**3.17**	IC_50_ 4.11 μg/mL (HSV-1)	(2*R*)-2-(2,3,6-tribromo-4,5-dihydroxybenzyl)- cyclohexanone [84]

a Notes: *Staphylcoccus aureus* ATCC29213;

b *Staphylcoccus aureus* 02–60;

c *Staphylcoccus epidermidis* ATCC12228;

d *Staphylcoccus epidermidis* 02–4;

e *Escherichia coli* ATCC25922;

f *Pseudomonas aeruginosa* ATCC27853;

g *Pseudomonas saeruginosa* 02–29,

h MC155 strain of *Mycobacterium smegmatis*;

i mean bacteriocidal and fungicidal MIC;

j mean bacteriostatic and fungistatic MIC.

**Table 4:** IC_50_ for enzyme inhibition and names of compounds in Scheme 4.

No.	IC_50_	Names
**4.1**	2.4 a	2,2′,3,3′-tetrabromo-4,4′,5,5′-tetra-hydroxydiphenyl methane [70]
**4.2**	1.7 a	3-bromo-4,5-bis(2,3-dibromo-4,5-dihydroxybenzyl)pyrocatechol [70]
**4.3**	1.5 a 0.098 b	Bis(2,3-dibromo-4,5-dihydroxybenzyl)ether [8,10,11,70]
**4.4**	0.84 a	2,2′,3-tribromo-3′,4,4′,5-tetrahydroxy-6′-ethyloxymethyldiphenylmethane [70]
**4.5**	0.03 b	Bis(2,3,6-tribromo-4,5-dihydroxybenzyl)ether [8]
**4.6**	100 b	3-bromo-4,5-dihydroxybenzylalcohol [8]
**4.7**	25 b	4-bromo-2,3-dihydroxy-6-hydroxymethylphenyl 2,5-dibromo-6-hydroxy-3-hydroxy-methylphenyl ether [11]
**4.8**	11 b	2,3,6-tribromo-4,5-dihydroxybenzylalcohol [10]
**4.9**	89 b	2,3-dibromo-4,5-dihydroxybenzylalcohol [11]
**4.10**	110.4 b	2,4-dibromophenol [7]
**4.11**	60.3 b	2,4,6-tribromophenol [7]
**4.12**	0.11 c	2,2′,3,6,6′-pentabromo-3′,4,4′,5-tetrahydroxydibenzyl ether [31]
**4.13**	0.4 c	Bis(2,3,6-tribromo-4,5-dihydroxyphenyl)methane [31]
**4.14**	0.4 c	2,2′,3,5′,6-pentabromo-3′,4,4′,5-tetrahydroxydiphenylmethane [31]
**4.15**	1.15 c	2,3,6-tribromo-4,5-dihydroxymethylbenzene [31]
**4.16**	0.25 c	2,3,6-tribromo-4,5-dihydroxybenzaldehyde [31]

a Notes: IC_50_ (μM) for PTP1B inhibition;

b IC_50_ (μM) for α-glucosidase inhibition;

c IC_50_ (μg/mL) for aldose reductase inhibition.

## Abbreviations

BPs: bromophenols
SAR: structure and activity relationship
T2DM: type 2 diabetes mellitus
PTP1B: protein tyrosine phosphatase-1B
HMG-CoA: 3-hydroxy-3-methylglutaryl coenzyme A
MIC: minimum inhibitory concentration
MCF-7: human breast adenocarcinoma cell line
KB: human carcinoma of the nasopharynx cell line
DLD-1: colorectal adenocarcinoma cell lines
HCT-116: human colon carcinoma cells
HCT-8: human epithelial intestinal cell line
Bel-7402: human hepatoma cell line
BGC-823: human gastric carcinoma cell line
A549: human lung adenocarcinoma epithelial cell line
A2780: human ovarian carcinoma
SH-SY5Y: neuroblastoma cell line
PC12: neuroendocrine cells
HELF: human embryo lung fibroblasts
HSV-1: herpes simplex type 1
AP^r^ HSV-1: phosphonoacetic acid-resistant HSV-1
TK^−^HSV-1: thymidine kinase deficient HSV-1
IHNV: infectious hematopoietic necrosis virus
IPNV: infectious pancreatic necrosis virus
